# Default mode and fronto-parietal network associations with IQ development across childhood in autism

**DOI:** 10.1186/s11689-022-09460-y

**Published:** 2022-09-15

**Authors:** Joshua K. Lee, An Chuen Billy Cho, Derek S. Andrews, Sally Ozonoff, Sally J. Rogers, David G. Amaral, Marjorie Solomon, Christine Wu Nordahl

**Affiliations:** 1 MIND Institute, University of California Davis School of Medicine, Sacramento, CA USA; 2 Department of Psychiatry and Behavioral Sciences, University of California Davis School of Medicine, Sacramento, CA USA; 3 Department of Radiology, University of California Davis School of Medicine, Sacramento, CA USA

**Keywords:** Autism spectrum disorder, Intellectual disability, IQ, MRI, Longitudinal, Default mode, Fronto-parietal

## Abstract

**Background:**

Intellectual disability affects approximately one third of individuals with autism spectrum disorder (autism). Yet, a major unresolved neurobiological question is what differentiates autistic individuals with and without intellectual disability. Intelligence quotients (IQs) are highly variable during childhood. We previously identified three subgroups of autistic children with different trajectories of intellectual development from early (2–3½ years) to middle childhood (9–12 years): (a) persistently high: individuals whose IQs remained in the normal range; (b) persistently low: individuals whose IQs remained in the range of intellectual disability (IQ < 70); and (c) changers: individuals whose IQs began in the range of intellectual disability but increased to the normal IQ range. The frontoparietal (FPN) and default mode (DMN) networks have established links to intellectual functioning. Here, we tested whether brain regions within the FPN and DMN differed volumetrically between these IQ trajectory groups in early childhood.

**Methods:**

We conducted multivariate distance matrix regression to examine the brain regions within the FPN (11 regions x 2 hemispheres) and the DMN (12 regions x 2 hemispheres) in 48 persistently high (18 female), 108 persistently low (32 female), and 109 changers (39 female) using structural MRI acquired at baseline. FPN and DMN regions were defined using networks identified in Smith et al. (Proc Natl Acad Sci U S A 106:13040–5, 2009). IQ trajectory groups were defined by IQ measurements from up to three time points spanning early to middle childhood (mean age time 1: 3.2 years; time 2: 5.4 years; time 3: 11.3 years).

**Results:**

The changers group exhibited volumetric differences in the DMN compared to both the persistently low and persistently high groups at time 1. However, the persistently high group did not differ from the persistently low group, suggesting that DMN structure may be an early predictor for change in IQ trajectory. In contrast, the persistently high group exhibited differences in the FPN compared to both the persistently low and changers groups, suggesting differences related more to concurrent IQ and the absence of intellectual disability.

**Conclusions:**

Within autism, volumetric differences of brain regions within the DMN in early childhood may differentiate individuals with persistently low IQ from those with low IQ that improves through childhood. Structural differences in brain networks between these three IQ-based subgroups highlight distinct neural underpinnings of these autism sub-phenotypes.

**Supplementary Information:**

The online version contains supplementary material available at 10.1186/s11689-022-09460-y.

## Background

Intellectual functioning, as assessed using standardized intelligence quotients (IQ) or developmental quotients (DQ), is highly variable in autistic^1^ children. The CDC estimates that 33% of autistic children have IQs in the range of intellectual disability (IQ < 70), 24% in the borderline IQ range of 70–85, and 42% with average or higher IQs (>85) [4]. The trajectory of intellectual functioning across childhood is also highly variable [5–7]. For example, we previously investigated the change in IQ scores from 3 to 8 years of age and identified four autistic subgroups: two groups, comprising 26% and 18% of the sample, respectively, had mean IQs in the intellectual disability range at both time points (one lower, one higher); a third group (22%) had IQs in the average range or better at both time points. Of particular interest was a fourth group (which we called “changers”), comprising roughly one third of the cohort (35%). This changers group began the study with IQs in the range of intellectual disability but made significant gains (average of 34 points) to have IQs in the average range by ages 6–8 [8].

One of the major unresolved questions concerning the neurobiology of autism is what differentiates autistic individuals with and without intellectual disability. While there have been hundreds of MRI studies of brain organization in autism, an incredibly small number have examined children with IQs below 85. A 2016 query of the National Database for Autism Research found that out of 47,400 total participants with autism, only 11% had IQs less than 85 and <1% of these had neuroimaging data available [9]. Accordingly, autistic individuals with intellectual disabilities are understudied, and very little is known about if and how their brains develop differently from autistic children without ID.

One notable exception is Reiter et al. [10] who found resting state fMRI patterns of network organization were different between groups of autistic children (6–15 years) with IQ < 85 and IQ > 115. The group with lower IQ demonstrated significant underconnectivity within the default mode network (DMN) and within the ventral visual stream. The participants with higher IQ had reduced network segregation compared to typically developing controls (TD). Gabrielsen et al. [11] studied 7–17-year-old autistic children and reported that autistic children with low verbal and cognitive performance had decreased within-network functional connectivity in default, salience, auditory, and frontoparietal networks and decreased interhemispheric functional connectivity than autistic children with normal verbal ability and cognitive performance.

Historically, investigations of the neural bases of intelligence have focused on positive correlations between total brain volume and intellectual functioning. This positive relationship has been reported both within the normative range of IQ [12, 13] and within the range of intellectual disability [14]. Voxel-based morphometry studies of gray matter density have associated higher IQ with increased gray matter densities across frontal, temporal, and posterior cingulate cortices [15]. Other neuroimaging research has focused on the fronto-parietal network (FPN) (also referred to as the central-executive network). Structural differences in the FPN are associated with aspects of working memory [16], and both structural and functional differences in the FPN are associated with response inhibition and set shifting [17]; these kinds of cognitive operations support fluid intelligence [18–20]. Consequently, the FPN forms the basis of a prominent conjecture, the parieto-frontal integration theory of intelligence [21]. More recently, functional connectivity of the default mode network (DMN) has also been implicated in intellectual functioning [22]. In particular, the magnitude and quality of functional interactions between the DMN and FPN correlate with individual differences in IQ [22–27]. Although the DMN and FPN are often evaluated using resting-state functional scans, structural covariance studies have shown that gray matter volumes within the brain regions comprising the FPN and DMN networks covary [28, 29], consistent with developmental relationships joining structure and function in the brain [30].

In an extension of our previous work [8], which had identified IQ trajectory subgroups through 8 years of age in the UC Davis MIND Institute Autism Phenome Project, we recently reassessed IQ trajectories through 12 years of age using latent class growth analysis [31]. We identified three groups: those with (1) IQ scores in the normal range across childhood (persistently high IQ; P-high), (2) IQ scores in the range of intellectual disability across childhood (persistently low IQ; P-low), and (3) a group with low IQ in early childhood that made IQ gains that plateaued by age 12 (changers) [31].

In the current study, we assessed associations between the volumes of anatomically defined regions within the FPN and the DMN at baseline (~3 years of age) between the IQ trajectory groups using multivariate distance matrix regression (MDMR) [32]. MDMR tests for associations between phenotypic variables (e.g., age, sex, IQ group) and a distance matrix––a pair-wise matrix indicating the degree to which individuals differ across a set of measurements, in the present case, volumes of regions of interest in the FPN and DMN. Complementing this analysis, we conducted MDMR effect-size analysis to assess which brain regions within the FPN and DMN contributed the most to group differences [33]. Prior evidence suggests that there are functional connectivity differences between autistic children with and without ID in the FPN and DMN [10]. On the basis that functional connectivity and volumetric brain structure are developmentally related [30], we predicted that we would observe volumetric differences in FPN and DMN brain regions across the three IQ-trajectory subgroups. Specifically, we hypothesized that differences in FPN and DMN structure at baseline are predictive of change in IQ scores across development and would differ between the changers and P-low IQ groups relative to the P-high group.

## Methods

### Participants

The current study includes 265 participants (89 females/176 males) diagnosed with an autism spectrum disorder. Intellectual ability was assessed at up to three time points (time 1: M(SD)=3.2 (0.5) years; time 2: 5.4 (1.0) years; time 3: 11.3 (0.5) years). At time 1 participants were assessed using the Mullen Scales of Early Learning (MSEL) [34] which provides developmental quotients (DQ). At time 2 and time 3 either the MSEL or the Differential Ability Scale (DAS) [35] was administered, depending on language ability. A longitudinal analysis of IQ was based on either the MSEL DQ score or the DAS General Conceptual Ability Standardized Score (GCA SS); each of these standardized scores has a mean of 100 and a standard deviation of 15. Demographic information, including family income and parental education, was collected at baseline.

Autism was diagnosed by research–reliable clinical psychologists at the UC Davis MIND Institute using the Autism Diagnostic Interview–Revised and the Autism Diagnostic Observation Schedule (ADOS)-Generic or ADOS-2 [36–39]. Participants were English-speaking without suspected vision, hearing, or neurological conditions. All research activities were conducted at the University of California Davis MIND Institute and Imaging Research Center and were approved by the UC Davis Institutional Review Board. Parents or legal guardians provided informed consent prior to participation. The data described in the current research is available from the corresponding or senior authors upon reasonable request.

### Identification of IQ trajectory groups through middle childhood

Autistic individuals were grouped according to IQ trajectories based on recent findings [31]. Three IQ trajectory groups were identified via latent class growth analysis [26]: (1) persistently high IQ (P-high; *n*=59), individuals whose IQs remained within normal range throughout childhood; (2) persistently low IQ (P-low; *n*=167), individuals whose IQs remained low in the range of intellectual disability throughout childhood; and (3) changers (*n*=147): individuals whose IQs began in the range of intellectual disability but increased over childhood. Mean IQs of each group at baseline and outcome time points are reported in Table 1, as well as other sample characteristics. A full description of the analysis can be found in Supplement 1.

**Table 1:** Sample Characteristics of Autistic Cohort

	Persistent High (*n* = 48)		Persistent Low (*n* = 108)		Changers (*n* = 109)	
	Males	Females	Males	Females	Males	Females
Participants	30	18	76	32	70	39
Baseline DQ/IQ	97.8 (12.3)	97.3 (11.8)	46.5 (10.7)	44.0 (12.7)	66.5 (11.1)	70.3 (11.1)
Outcome DQ/IQ	106.0 (16.9)	106.0 (15.7)	46.5 (12.4)	40.2 (13.8)	84.3 (18.5)	79.3 (15.9)
Baseline ADOS CSS	6.8 (2.0)	6.3 (1.7)	7.9 (1.7)	8.4 (1.3)	7.2 (1.6)	7.0 (1.3)
Baseline Total Brain Volume (cm^3^)	1,063 (68)	1,011 (90)	1,087 (103)	1,001 (112)	1,072 (93)	1,011 (121)

Means *(SD)* are reported; *DQ/IQ* Developmental /Intelligence Quotient, *ADOS CSS* ADOS Calibrated Severity Score

### Imaging acquisition and processing

Structural MRI at time 1 was acquired during natural nocturnal sleep [40] on a 3 Tesla Siemens Trio with an 8-channel head-coil (T1-weighted MPRAGE. TR 2170 ms, TE 4.86 ms, FOV 256, 192 sagittal slices, 1.0-mm slice thickness, 8:46 acquisition time). Spatial inhomogeneity in images was distortion corrected using a calibration phantom acquired at each scan (ADNI MAGPHAM, The Phantom Laboratory; Image Owl, Inc., Greenwich, NY, USA, http://www.phantomlab.com) [41]. Images were visually assessed for the quality using previously described quantitative procedures [42]. Overall, success rates for obtaining MRI scans were high (>86%). The changers group had a moderately higher success rate than the persistently low IQ group (94 vs 86%, *z*=2.1, se=.42, *p*=.033); success rates of other group comparisons were not significant (see supplement 2 for details of scanning success rates).

Identifiable information was removed from each MPRAGE, defaced, and then uploaded to MRICloud (https://mricloud.org) [43] for image segmentation. Gray and white matter labels defined by the LONI Probabilistic Brain Atlas protocol (LPBA40) [44, 45] were segmented using multi-atlas image segmentation. Multi-atlas image segmentation registers a set of age-appropriate anatomically labeled atlases onto a target brain image using diffeomorphic registration, which produces a set of candidate segmentations, and finally, consensus segmentations are produced using joint-label fusion, a voting procedure [46–48]. The authors have used these advanced segmentation routines in previous empirical publications [49–51].

### Network definitions

Regions of interest (ROI) prototypical to the DMN and FPN were identified by overlaying the published intrinsic functional connectivity group maps from Smith et al. [52] onto a standardized brain and corresponding segmented image. The statistical maps of the DMN and bilateral FPN (Network 4, 9–10 from [52]) were thresholded at *z* ≥ 2.3. ROIs with complete overlap with the thresholded network maps were included. ROIs with partial overlap were included if at least 75% of the voxels within the ROI were contained within the network map at the *z*=2.3 threshold and if the remaining 25% of voxels fell within the DMN/FPN network maps from [52] defined by a more lenient statistical threshold (*z* ≥ 1.7).

#### FPN network

The FPN comprised the following bilateral ROIs: superior frontal gyrus (prefrontal cortex), middle frontal gyrus (posterior segment and dorsal prefrontal cortex), pars opercularis and pars orbitalis of the inferior frontal gyrus, precentral gyrus, supramarginal gyrus, angular gyrus, precuneus, inferior temporal gyrus, and the middle occipital gyrus.

#### DMN network

The DMN comprised the following bilateral ROIs: posterior cingulate cortex, rostral anterior cingulate cortex, precuneus, cuneus, middle temporal gyrus, angular gyrus, gyrus rectus, middle frontal orbital gyrus, the prefrontal and poles of the superior frontal gyrus, amygdala, and parahippocampal gyrus.

### Analytic strategy

Multivariate distance matrix regression (MDMR) was used to identify associations between IQ trajectory groups and volumes of ROIs within the FPN and DMN networks at time 1. MDMR is a robust, person-centric, multivariate regression method with application in connectomic, genomic, and ecological research [32, 53, 54]. MDMR regresses a Gower-transformed pair-wise distance matrix onto an explanatory model and residual term [32, 55]. The distance matrix is an index representing how much individuals differ from one another across a set of outcomes (e.g., brain measurements). When constructing a distance *matrix*, a distance *metric* must be chosen. Here, we chose the Manhattan “city-block” distance, as we have done previously [53], because it is more robust to extreme values than either Euclidean and Pearson’s distances, and can be a better choice for high-dimensional datasets [56].

Interpretation of the results of MDMR regressions is aided by distance-based redundancy analysis, a data-reduction ordination technique that constructs new axes comprising linear combinations of predictors of interest (e.g., IQ trajectory groups) that best explain variation in the distance matrix [57, 58], and by using Euclidean projection to estimate exact distances between group centroids in multivariate space [59]. Finally, we use a resampling procedure (jack-knife) to estimate effect sizes for individual outcomes (e.g., brain regions within the network) to each MDMR predictor [33, 53]. In brief, given predictors (*X*_n×p_) associated with the multivariate outcome (*Y*_n×q_), the effect sizes *δ* of each individual outcome comprising *Y* is estimated by systematically dissociating each outcome of *Y* from the predictors *X* via permutation (shuffling the elements of *Y*). In each permutation, a new distance matrix *D*_*k*_ is computed from each shuffled *Y*_*k*_ and regressed onto *X*. Effect sizes are computed by estimating the change in the pseudo-*R*^2^ statistic (conceptually similar to the change in *R*^2^ in linear models) that results after comparing the pseudo-*R*^2^ of the permuted and unpermuted regressions. Critically, effect sizes are relative and scale with dimensionality and covariance of *Y*, and thus, *δ* should not be compared between studies.

Analyses proceeded through three steps: (1) identify the overall pattern of differences between groups (P-high, P-low, changers) separately across the FPN and DMN, (2) identify the specific brain regions within the network that contribute most to the multivariate difference using effect-size analysis, and (3) descriptively assess the nature and extent of volumetric differences between groups in ROIs with the largest effect sizes using univariate linear regression.

All analyses employed the following model: volume(s) = *β*_0_ + *β*_1_ (IQ trajectory groups) + *β*_2_(age) + *β*_3_(sex) [model 1]. Interactions between sex and trajectory groups were also tested. The volumes of each brain region were converted into proportions of the participant’s total brain volume, controlling for overall brain-size differences. The proportions for each brain region were then T-scored across participants to account for overall size differences between brain regions. We also investigate the impact of parental annual income [model 2] and educational attainment [model 3] covariates on group differences in the FPN and DMN.

## Results

### Sample characteristics

Table 1 reports sample characteristics. Baseline ADOS calibrated severity scores (CSS) were significantly higher in P-low group compared to changers (*b*=.89, SE = .22, *p* < .0001) and the P-high group (*b*=1.4, SE = .29, *p* < .0001). ADOS CSS scores did not differ significantly between changers and the P-high group (*b*=.5, SE = .30, *p* = .089). Baseline DQ scores differed between all three groups (*p* < .0001). Baseline total brain volume did not differ between trajectory groups, *F*(2, 141) = 1.62, *p* = 0.20). Trajectory groups did not significantly differ in parental annual income (*χ*^2^(12) = 13.5, *p* = 0.34) or educational attainment (*χ*^*2*^(6) = 10.1, *p* = 0.12) at baseline; see Supplements 3 and 4, respectively.

### MDMR analysis

#### FPN network

**Fig. 1:**
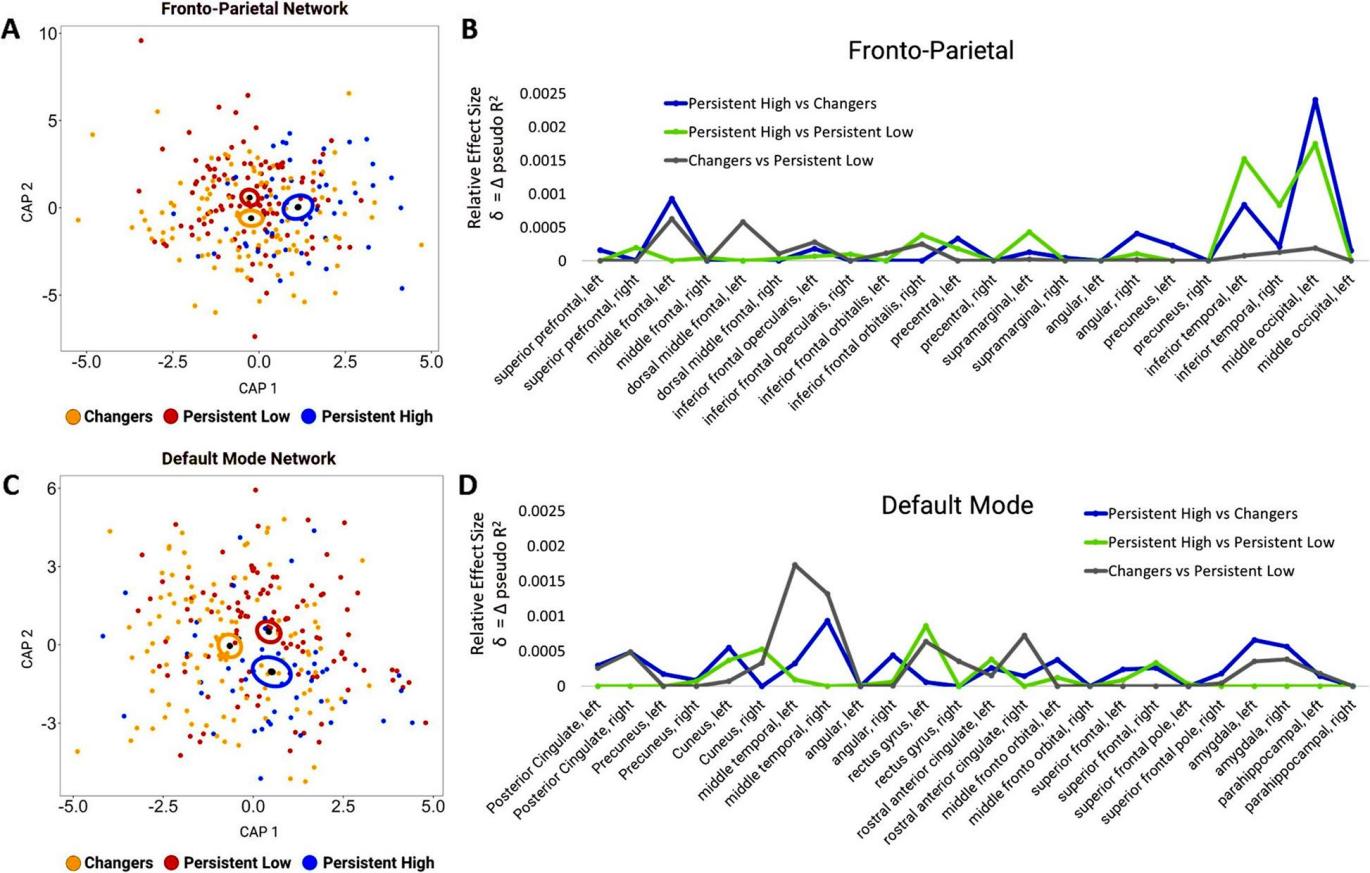
Depictions of results from analyses of regional volumes from the fronto-parietal network (FPN) and default mode network (DMN) networks using multivariate distance matrix regression (MDMR) and effect size analysis. Ordination plots depict the significant MDMR differences between longitudinal IQ trajectory groups in the **a** FPN and **b** DMN. These plots were produced using distance-based redundancy analysis (dbRDA) to facilitate visualization of high-dimensional data. The black dots depict median centroid locations of the changers, persistent-low, and persistent-high groups on the first two dbRDA axes. The ellipsoids indicate the standard error of the locations of each group centroid on those two axes. Results of effect-size analyses of the **c** FPN and **d** DMN are depicted for the pair-wise differences between the changers, persistent low, and persistent high groups

MDMR analysis demonstrated that FPN volumes in both the changers (*β* = .008, *p* = .010) and P-low (*β* = .008, *p* = .009) groups significantly differed from the P-high group, but that the P-low and changers did not significantly differ from each other (*β* = .003, *p* = .721) (Fig. 1A) (Table 2, model 1). Regional effect size analysis underlying these group differences is reported in Fig. 1B. The two largest effect sizes underlying the difference between changers and P-high groups were the left middle occipital gyrus and left inferior temporal gyrus. Post hoc regressions indicate that left middle occipital gyrus volumes were smaller (*β* = −0.65, SE = 0.17, *p* = .0002) and left inferior temporal gyrus volumes were larger (*β* = 0.44, SE = 0.17, *p* = .011) in changers compared to the P-high groups. The two largest effect sizes underlying differences between P-low and P-high groups were also in the left middle occipital gyrus and left inferior temporal gyrus. Post hoc regressions indicated that left middle occipital gyrus volumes were smaller (*β* = −0.56, SE = 0.17, *p* = .001) and left inferior temporal gyrus volumes were larger (*β* = 0.58, SE = 0.17, *p* = .0009) in the P-low group compared to the P-high group.

**Table 2:** Paired Comparisons from Multivariate Distance Matrix Regressions

Network	Effect	Model 1 (Age + Sex + Group) *p*-value	Model 2 (Age + Sex + Group + Annual Income) *p*-value	Model 3 (Age + Sex + Group + Parental Education) *p*-value
Fronto-Parietal	Persistent High vs Persistent Low	0.009	0.018	0.034
	Persistent High vs Changers	0.010	0.027	0.063
	Changers vs Persistent Low	0.721	0.631	0.693
Default Mode	Persistent High vs Persistent Low	0.391	0.412	0.27
	Persistent High vs Changers	0.023	0.041	0.27
	Changers vs Persistent Low	0.017	0.019	0.27

Biological sex also differentiated FPN volumes in autistic individuals (*β* = .009, *p* = 0.003), although interactions between sex and trajectory groups were not significant (*β* s ≤ .005, *p*s ≥ 0.166). Effect sizes underlying the sex differences within the FPN are reported in Supplemental 5A. The two largest effect sizes were exhibited within the right angular and right middle frontal gyri. Post hoc regressions indicate that these regions were larger in autistic females than in autistic males (*βs* ≥ 0.29, *p*s ≤ .01). Table 1 reports the *p* values of group differences after inclusion of annual [model 2] and parental educational attainment [model 3] covariates. In the FPN, annual income and parental education did not substantively alter group differences.

#### DMN network

MDMR analysis demonstrated that both the P-low (*β* = .008, *p* = .017) and P-high groups (*β* = .007, *p* = .023) significantly differed from the changers group in DMN volumes, but the P-low and P-high groups did not significantly differ (*β* = .004, *p* = .391) (Fig. 1C) (Table 2). Effect size analyses are reported in Fig. 1D. The two largest effect sizes underlying the difference between changers and P-low groups were the left and right middle temporal gyri. Post hoc regressions indicate that both left and right gyri were smaller in changers (*βs ≤* −0.35, SE = 0.13, *ps ≤* .018). The two largest effect sizes underlying the difference between changers and P-high groups were right middle temporal gyrus and left amygdala volumes. Post hoc regression indicates that both were smaller in changers (*βs ≤* −0.39, SE = 0.17, *ps ≤* .025). We note that the effect sizes distinguishing changers and P-high groups were relatively more diffuse than was seen in other group comparisons.

Biological sex-differentiated DMN volumes in autistic individuals (*β* = .007, *p* = 0.03). However, sex did not significantly interact with the trajectory group (*Bs* ≤ .004, *p*s ≥ 0.498). Effect sizes underlying the sex differences are reported in Supplemental 5B. The two brain regions with the largest effect sizes were right angular and right parahippocampal gyri in females. Post hoc regressions indicated that these regions were larger in females than in males (βs ≥ 0.29 *Z* score, *p*s ≤ 0.025). Finally, we note that the angular gyri are constituents of both the FPN and DMN, suggesting a potentially interesting site for future studies of sex differences. Familial annual income and parental educational attainment covariates did not substantively alter the pattern of results in the FPN. Family income also did not change the pattern of group differences observed in the DMN. However, the inclusion of parental education attenuated the significant group differences in the DMN, though was not by itself, a predictor of DMN volumes.

## Discussion

The present study utilized structural imaging to examine 3 empirically defined autistic subgroups with differential IQ developmental trajectories from 2 to 12 years of age. Our aim was to identify the neurobiology which differentiates subgroups with persistently high, persistently low, and improving IQ scores. Our multivariate analyses found differences between the P-high subgroup versus the other two subgroups (changers, P-low) in the FPN and differences between the changers subgroups versus the other two subgroups (P-high, P-low) in the DMN. These findings support our hypothesis that volumetric differences in brain regions of the FPN and DMN at baseline may contribute to the differentiation of IQ trajectories.

### Networks supporting intellectual ability and its impairment

The literature of the neural bases of intelligence has long focused on the functional activity of the FPN (also referred to as the central-executive network) [22], a task-positive functional network that exhibits increased BOLD activation and connectivity with increasing cognitive load across a wide array of tasks involving multiple demands [22, 60, 61]. More recently, there has been growing interest in the role of the DMN in supporting general intellectual functioning. In contrast to the FPN, the DMN is a task-negative functional network whose activations tend to be anti-correlated with those of the FPN, the strength of which has been associated with individual differences in IQ measures [23–25]. Large numbers of imaging studies have examined the structural and functional neural correlates of intellectual functioning within the normal range [21]. Several studies have identified associations between functional activations in regions within the FPN and IQ in autistic children without intellectual disabilities [62, 63]. Studies that include autistic children with intellectual disabilities are limited [10, 11]. The extant non-autism research frequently reports altered functional connectivity between the FPN and DMN in association with intellectual impairments, as well as structural alterations to gray matter density in the dorsomedial prefrontal cortex and other regions within the FPN [64]. However, these studies have largely focused on specific patient populations, for example, Down Syndrome and Williams Syndrome [65].

Here, we found that at time 1 (~3 years of age), prior to changes in IQ, both the P-low and changers groups differed from the P-high group in the volumes of left inferior temporal and left middle occipital gyri of the FPN. These regions are broadly implicated in supporting language, semantic knowledge, and sensory integration, including visual information and object recognition [66, 67]. This suggests that low IQ in early childhood may be associated with alterations to processes of sensory and perceptual integration.

In the DMN network, both P-low and P-high groups differed from the changers group, indicating that the DMN may be involved in developmental compensatory mechanisms. The middle temporal gyrus appears to be the key hub region, as indicated by its prominence in the effect-size analyses. The middle temporal gyri are also notable in that they too subserve language function, semantic knowledge processing, and sensory integration [66, 67].

### Research and clinical implications

Intellectual disability and other psychiatric and physiological comorbidities do not occur evenly in the autism population, suggesting the presence of autism subtypes with distinct clinical profiles and etiologies [68]. In our recent work [31], we demonstrated that the three identified IQ trajectory autism subgroups also presented unique trajectories in autism symptom severity, adaptive functioning, and internalizing and externalizing characteristics. It may be possible that the present findings not only serve as evidence of neurobiological differences between the IQ trajectory subgroups, but also as endophenotypes linked to the expression and severity level of clinical characteristics [69]. As such, this study adds a piece of neurobiological evidence to the growing literature which suggests associations between autism and intellectual disability are accompanied by variation in brain structure [70]. Accordingly, this study also contributes to the distal goals of early clinical screening and intervention, as the identification of early brain markers of IQ trajectories could potentially provide useful prognostic information to parents and other caregivers that could be used to guide subsequent treatment or provide clues regarding the etiology of autism and autistic sub-phenotypes.

### Limitations

The present research has several strengths, including the use of empirically extracted subgroups based on longitudinal measurements of IQ in childhood to look at brain differences is a significant strength of this study, the use of a person-centric multivariate approach that allowed us to examine differences across whole networks. However, several limitations merit consideration. First, we only examined volumetric differences in brain structure between IQ trajectory groups at the time of study enrollment in early childhood. This was motivated by a desire to identify early neural predictors of subsequent behavioral change. Future research should examine the full relationship between concurrent changes in both the brain and behavior. Another limitation of our approach was that it did not allow for individual differences in the spatial topology of DMN and FPN canonical networks [52]. An alternative approach would be to uniquely identify each individual’s network topology using resting-state MRI. However, this approach presents additional limitations including, (a) test-retest reliability of these networks has intraclass correlation coefficients less than ~0.60 [71], indicating substantial test-retest variability that could dominate any downstream volumetric analysis, (b) more restricted sample sizes due to inherent difficulties in acquiring quality resting-state data, especially in 3-year-old children, and (c) it is unclear how individually different topologies could be compared volumetrically, other than by its total volume. In contrast, our approach allows for the straightforward analysis of group differences of regions within the group-level canonical network [52]*.* We also note that our individual regions of interest were defined by the LONI parcellation [43]; however, other parcellation schemes (e.g., functional parcellations) could have been employed, which may have potentially led to different results, depending on the optimal degree of regional specificity. The future research could also forgo parcellations in favor of a voxel- or vertex-wise approach, which could more precisely adhere to the exact boundaries of each canonical network. The sampling variability remains high even in moderately large datasets, which can contribute to poor replicability and/or inflated effect sizes [72, 73]; consortium-sized datasets may present an opportunity for future research. Finally, this study cannot completely rule out environmental confounds and interactions, an area ripe for future research.

## Conclusions

Here, we report structural brain differences in functionally defined networks associated with intellectual functioning in three empirically derived, IQ trajectory autistic subgroups. The DMN may be important in differentiating individuals with persistently low IQ from those with more transitory low IQ that improve to moderate IQ through childhood. These results are potentially indicative of successful compensatory processes which may be targeted by future interventions.

## Supplementary Information

**Additional file 1: Supplemental 1. Supplement 2.** Acquisition Success by IQ Trajectory Group. **Supplement 3.** Familial Annual Income at Baseline. **Supplement 4.** Highest Educational Attainment of One or More Primary Caretakers at Baseline. **Supplemental 5**.

## Data Availability

Data described in this research is available from the corresponding author upon reasonable request.

## Footnotes

1 Our use of identity-first over person-first terminology is motivated by members of the autism community [1–3].
