# Supplementary material for: Default mode and fronto-parietal network associations with IQ development across childhood in autism
Source: J Neurodev Disord. 2022 Sep 15;14:51. doi: 10.1186/s11689-022-09460-y (PMC9479280; doi:10.1186/s11689-022-09460-y)
Supplement: Supplementary file 1 — Additional file 1: Supplemental 1. Supplement 2. Acquisition Success by IQ Trajectory Group. Supplement 3. Familial Annual Income at Baseline. Supplement 4. Highest Educational Attainment of One or More Primary Caretakers at Baseline. Supplemental 5. [file 11689_2022_9460_MOESM1_ESM.docx]

**Supplemental 1**

In recent work (1), we identified three IQ trajectory groups: (a) Persistently-High IQ (P-High): Individuals whose IQs remained within normal range throughout childhood, n=48 (18 female), (b) Persistently-Low IQ (P-Low): Individuals whose IQs remained low in the range of intellectual disability throughout childhood, n=108 (32 female), and (c) Changers: Individuals whose IQs began in the range of intellectual disability but increased over childhood, n=70 (39 female). Briefly, trajectory groups had been identified using latent class growth analysis (2) to identify distinct IQ trajectories in a sample of ASD youth, with age matched TD controls serving as a reference group. All participants with at least one timepoint were included in the analysis (*n*_ASD_=373; *n*_TD_=162). Linear and quadratic age-based models were tested for the ASD group. Both information-heuristic (e.g., information criterion values) and inferential (e.g., likelihood ratio tests) relative fit comparisons were used to select the best-fitting modeling solution (3). Namely, information-heuristic indices include the Akaike Information Criterion (AIC) (4), Bayesian Information Criterion (BIC) (5), and sample size-adjusted BIC (SBIC) (6), in which lower values indicate better fit, as well as the approximate Bayes Factor (7,8). As an inferential index, the approximate correct model probability (CMP) (5) compares a single model versus all other models under consideration, and models with a CMP >.10 should be considered as candidate models.

| **Supplement 2.** Acquisition Success by IQ Trajectory Group | | | |
| --- | --- | --- | --- |
|  | Persistently Low | Changers | Persistently High |
| Success | 130 | 125 | 50 |
| Failure | 21 | 8 | 6 |
| Percent Success | 86% | 94% | 89% |
| Notes: Success Rate significantly differed between Persistently Low and Changers IQ trajectory groups (z=2.1, se=.42, p=.033). Other paired comparisons were not significant (*p*s ≥ .28). | | | |

| **Supplement 3. Familial Annual Income at Baseline** | | | | |
| --- | --- | --- | --- | --- |
|  | Changers (n=99) | Persistent High  (n = 40) | Persistent Low  (n = 88) | χ^2^ |
| Under $10 000 | 4 | 0 | 3 | χ^2^(12) = 13.5,  *p* = 0.34 |
| $10 000-$29 000 | 16 | 0 | 9 |  |
| $30 000-$49 000 | 12 | 5 | 12 |  |
| $50 000-$74 999 | 16 | 12 | 21 |  |
| $75 000-$99 999 | 18 | 10 | 14 |  |
| $100 000-$149 000 | 16 | 6 | 13 |  |
| $150 000 and above | 16 | 7 | 19 |  |
| Missing or Non-Response | 10 | 8 | 20 |  |

| **Supplement 4. Highest Educational Attainment of One or More Primary Caretakers at Baseline** | | | | |
| --- | --- | --- | --- | --- |
|  | Changers (n = 98) | Persistent High  (n = 41) | Persistent Low  (n = 91) | χ^2^ |
| High School | 19 | 4 | 12 | χ^2^(6) = 10.1,  *p* = 0.12 |
| Associate | 19 | 13 | 27 |  |
| Bachelor | 37 | 8 | 26 |  |
| Post-Bachelor | 23 | 15 | 26 |  |
| Missing or Non-Response | 11 | 7 | 17 |  |

**Supplemental 5**


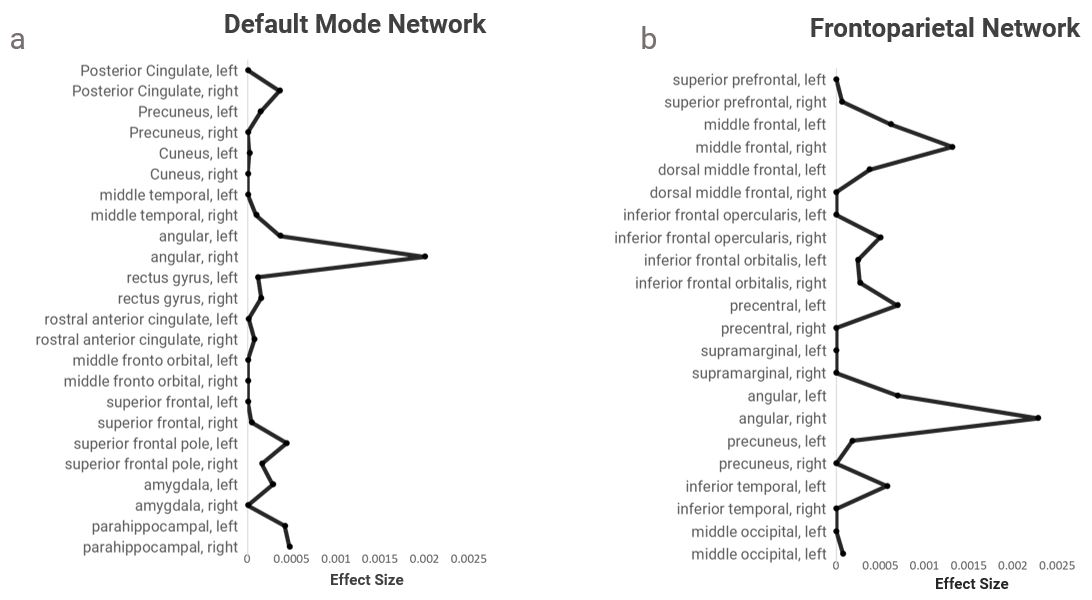


**Supplemental References**

1. Solomon M. IQ phenotypes of autistic youth from early childhood to preadolescence. In: Virtual Keynote. 2021.

2. Jung T, Wickrama K a. S. An Introduction to Latent Class Growth Analysis and Growth Mixture Modeling. Social and Personality Psychology Compass [Internet]. 2008 [cited 2021 Oct 10];2(1):302–17. Available from: https://onlinelibrary.wiley.com/doi/abs/10.1111/j.1751-9004.2007.00054.x

3. Masyn KE. Latent class analysis and finite mixture modeling. In: The Oxford handbook of quantitative methods: Statistical analysis, Vol 2. New York, NY, US: Oxford University Press; 2013. p. 551–611. (Oxford library of psychology).

4. Akaike H. A new look at the statistical model identification. IEEE Trans Autom Contr. 1974;19.

5. Schwarz G. Estimating the Dimension of a Model. The Annals of Statistics [Internet]. 1978 [cited 2021 Oct 10];6(2):461–4. Available from: https://www.jstor.org/stable/2958889

6. Sclove SL. Application of model-selection criteria to some problems in multivariate analysis. Psychometrika [Internet]. 1987 Sep [cited 2021 Oct 10];52(3):333–43. Available from: http://link.springer.com/10.1007/BF02294360

7. Diciccio TJ, Kass RE, Raftery A, Wasserman L. Computing Bayes Factors by Combining Simulation and Asymptotic Approximations. Journal of the American Statistical Association [Internet]. 1997 Sep 1 [cited 2021 Oct 10];92(439):903–15. Available from: https://doi.org/10.1080/01621459.1997.10474045

8. Wasserman L. Bayesian Model Selection and Model Averaging. Journal of Mathematical Psychology [Internet]. 2000 Mar 1 [cited 2021 Oct 10];44(1):92–107. Available from: https://www.sciencedirect.com/science/article/pii/S0022249699912786
